# In vitro screening as an anthelmintic discovery pipeline for *Calicophoron daubneyi*: nutritive media and rumen environment-based approaches

**DOI:** 10.1007/s00436-021-07066-2

**Published:** 2021-02-06

**Authors:** K. M. Huson, R. M. Morphew, A. Winters, A. Cookson, B. Hauck, P. M. Brophy

**Affiliations:** 1 Institute of Biological, Environmental & Rural Sciences (IBERS), Aberystwyth University, Aberystwyth, SY23 3DA UK; 2 Present Address: Agri-Food and Biosciences Institute (AFBI), Large Park, Hillsborough, BT26 6DR UK

**Keywords:** Paramphistomosis, *Calicophoron daubneyi*, Anthelmintic, Rumen, Oxyclozanide, Praziquantel

## Abstract

Paramphistomosis can lead to morbidity and mortality of ruminant livestock within tropical and sub-tropical climates. In recent decades, rumen fluke has become an emerging infection in temperate climates across Western Europe, with *Calicophoron daubneyi*, the primary species present. Clinical outbreaks with *C. daubneyi* larvae are reported and adults might be responsible for production losses. There is not currently a widely licensed anthelmintic product available to control *C. daubneyi.* In this study, three existing flukicide anthelmintics were tested for efficacy against mature *C. daubneyi*, comparing a standard in vitro culturing assay and a new more relevant rumen fluid based in vitro compound screening protocol. The new rumen based screen confirmed that oxyclozanide was active against adult *C. daubneyi* and identified activity with praziquantel. The study highlighted the downstream value of incorporating relevant in vitro screening for anthelmintic discovery pipelines.

## Background

Paramphistomosis is established as a significant cause of morbidity and mortality in ruminant livestock in tropical and sub-tropical climates (Godara et al. 2014; Rangel-Ruiz et al. 2003). In recent decades, paramphistome parasites have become an emerging infection of ruminant livestock in temperate climates across Western Europe (Ferreras et al. 2014; Mage et al. 2002; Gordon et al. 2013; Huson et al. 2017, 2018; Amalia Naranjo-Lucena et al. 2018; Sargison et al. 2019), with *Calicophoron daubneyi* clearly identified as the primary species present (Gordon et al. 2013; Jones et al. 2017; Ploeger et al. 2017; Zintl et al. 2014). A number of clinical cases attributed to acute paramphistomosis have also been reported (Mason et al. 2012; Millar et al. 2012). Infection, with adult paramphistomes can cause ruminal papillae atrophy and ulceration (Fuertes et al. 2015; Rolfe et al., 1994). The adult disease has been associated with production losses such as reduced milk yield and growth rates (Foster et al. 2008; Rojo-Vázquez et al. 2012), although the influence on production is not yet confirmed (Sargison et al. 2016).

At present, no widely licensed anthelmintic product is available to control either immature or mature paramphistome infections. The oxyclozanide-containing formulation Douvistome is registered in France for the control of *Paramphistomum* spp. and other oxyclozanide-containing products are reportedly used off-licence to treat paramphistome infections in countries where no licenced product is available. Oxyclozanide is the only commonly available anthelmintic compound for which efficacy against both the adult and juvenile life stages of rumen fluke infections has been consistently described (Paraud et al. 2009; Rolfe and Boray 1987). However, even at therapeutic doses, oxyclozanide may cause adverse effects including softening of the faeces, increased frequency of defecation and transient inappetence in cattle (VMD 2017), and reportedly impacts on body weight gain post-treatment (Shaheen et al. 2013). The effects of oxyclozanide overdose are significant, including depression, anorexia and diarrhoea at dosages >25 mg/kg BW in cattle and sheep (Swan 1999), and severe weight loss and death following doses of >60 mg/kg BW (Walley 1966). Meat and milk withdrawal periods of 13 and 4.5 days in cattle, and 14 and 7 days in sheep, respectively, must also be observed, making oxyclozanide a less than ideal choice of anthelmintic. Therefore, there is a clear need to investigate possible alternative anthelmintic and novel compound therapies.

Closantel (as closantel sodium dihydrate) has been suggested to reduce the output of rumen fluke eggs in cattle by in vivo FECRT (Arias et al. 2013). However, alternative research has demonstrated closantel to have no significant effect on reducing paramphistome egg outputs or parasite burden (Malrait et al. 2015; Rolfe and Boray 1987). Other compounds which have shown efficacy against juvenile and/or adult rumen fluke during in vitro and in vivo tests include niclosamide (Rolfe and Boray 1987), resorantel (Rolfe and Boray 1988) and bithionol sulfoxide (Prasitirat et al. 1996), but these compounds have largely been withdrawn from sale. Thus, oxyclozanide remains the only recommended compound for the control of paramphistomosis (Sanabria et al. 2014).

Specific flukicide discovery screens applied to fluke parasites in the past have typically involved screening live parasites, both adults collected from a definitive host and newly excysted juveniles obtained from infective metacercariae (Ibarra and Jenkins 1984; Pakharukova et al. 2015) in a simple nutritive media such as RPMI 1640 or DMEM, often supplemented with glucose as an energy substrate (Howe et al. 2015) and/or sera from various sources including chicken, faetal bovine and horse (McCusker et al. 2016; Wang et al. 2019). The in vitro maintenance of many parasitic helminths is problematic due to the complexity of their metabolism and inability to meet essential environmental conditions for their survival ex-host (Ahmed 2014). Parasites maintained in vitro are exposed to an environment less suited to their survival, and maintenance times are often limited as a consequence (Behnke et al. 2008). The maintenance of *Fasciola hepatica* juveniles in vitro over a 6-month period where development towards the adult phenotype was shown represents an example of successful optimisation for long-term studies (McCusker et al. 2016).

In consideration of rumen fluke, and the diverse rumen environment in which they reside, essentially an anaerobic fermentation chamber comprising of ingested feed material and saliva, and a rich microbial community including bacteria, fungi and protozoans, this study incorporated a more targeted in vitro screening assay for discovering compounds with activity against adult *C. daubneyi* within an environment more closely mimicking their natural niche environment. Three existing flukicide anthelmintics were tested for efficacy against adult *C. daubneyi.* The selected anthelmintics were praziquantel, oxyclozanide and closantel (as closantel sodium dihydrate). The compounds were evaluated in two different in vitro screening protocols (maintenance in a standard chemical media and maintenance in natural rumen fluid collected perimortem from cattle). Both oxyclozanide and closantel halogenated salicylanilides and act as anthelmintics by disrupting the pathway of oxidative phosphorylation in susceptible helminth species (Swan 1999). Praziquantel is believed to act by causing calcium ion influx and spasmodic muscle contraction (Vale et al. 2017) leading to paralysis of affected parasites, leaving them open to immune-mediated attack. The three compounds selected have previously been shown to have direct effects on trematode parasites, without the need for host metabolism for activation, as is the case with the flukicide triclabendazole (TCBZ), a key anthelmintic for treating fascioliasis. TCBZ is metabolised in the host liver by sulphoxidation or hydroxylation into a number of TCBZ metabolites, predominantly TCBZ sulphoxide (TCBZ–SO) and TCBZ sulphone (TCBZ–SO_2_) which are active against fasciolid parasites (Davis et al. 2020). Selecting only anthelmintics which have direct effects on their target trematode parasites was deemed important in this instance as mature rumen fluke have only a superficial attachment to the host and are, therefore, less likely to encounter metabolised forms of anthelmintics transported via the host bloodstream in significant quantities.

## Methods

### Test concentrations for known anthelmintics

Anthelmintic concentrations to test in both DMEM and rumen fluid maintenance conditions were selected based on the manufacturer’s recommended dose rates and existing literature on effective in vitro dosages for different parasitic flatworms. This was used to make an approximation of the maximum concentration which would be generated in the bovine rumen with respect to an oral dosing at the recommended in vivo dose. 150 l was assumed to be the average volume of a bovine rumen (Frandson et al. 2003). Dose rates of approximately 15 mg/kg bodyweight have been recommended for the treatment of paramphistomosis in cattle (Rolfe and Boray 1987). However, the manufacturers of the singular oxyclozanide-only drench marketed in the UK, Zanil (MSD Animal Health), recommend a maximum dosage in cattle of 105 ml, with an oxyclozanide concentration of 34 mg/ml. This equates to a maximum of 3.57 g of oxyclozanide per bovine and to a maximum rumen concentration of 60 μM generated for the standard maximum recommended dose of 105 ml, assuming a 150-l volume.

For closantel, the selected range of drug concentrations used was up to 75 μM, the same concentration as has previously been seen to be a rapidly (<2 h) effective dose against adult *F. hepatica* specimens in vitro, and was the highest tested dose in this previous experiment (Skuce and Fairweather 1990). Praziquantel is the frontline drug for treating human schistosomiasis (Chai 2013) and is currently used in veterinary medicine to control infections with tapeworms, and in tropical areas to control bovine schistosomiasis (McKellar and Jackson 2004; Olveda et al. 2016). The recommended in vivo dose for praziquantel to control schistosomiasis in cattle is 25 mg/kg (Olveda et al. 2016) which equates to a maximum potential rumen concentration of 320 μM in a 600 kg animal (assuming a 150-l volume). All the anthelmintics were dissolved in dimethyl sulfoxide (DMSO). The maximum final concentration of DMSO in any condition did not exceed 0.64% v/v.

### DMEM in vitro screen

Adult *C. daubneyi* were collected from naturally infected cattle identified in the slaughterhouse and transported directly to the laboratory in warm 39 °C PBS. Flukes were then added to 5 ml of DMEM media in 6-well culture plates (Corning® Costar® cell culture plates, Sigma, UK), 1 fluke/ml. Each well contained a fixed concentration of anthelmintic or solvent control (maximum volume of DMSO added to any well), or no additional treatment. Plates were then incubated at 39 °C and parasite motility scored (5 = normal activity to 0 = no activity; Table 1, based on Behnke et al. (2008)) hourly for 6 h. Media were then removed, 5 ml of fresh DMEM added and fluke maintained for a further hour before a final motility score was taken. In vitro maintenance was not extended beyond 7 h in the DMEM screen as previous work (data not shown) had identified that all untreated/control fluke did not reliably survive in DMEM for periods longer than 7–8 h. Flukes were then snap frozen for storage at −80 °C.

**Table 1:** Motility scoring chart based on Behnke et al. (2008) used to evaluate fluke activity during in vitro screens. Each individual well/tube was observed for approximately 30 s at each time point. Where stimulus was applied, this was touching with forceps in the case of DMEM treatment, and gentle tapping of the sealed tube in the case of the rumen fluid treatment

Motility score	Description
5	Normal activity, highly active ‘searching’ appearance to movement
4	Movement as normal but reduced frequency/speed
3	Slower/minimal movements, longer periods of inactivity
2	Little autonomous movement and smaller range, do respond to stimulus
1	Very little movement, examples such as a twitch in muscle only, only on response to stimulus
0	No movement observed

### Rumen fluid in vitro screen

Rumen fluke parasites from naturally infected cattle were collected in a local abattoir. Immediately before parasite collection, approximately 40 ml of fresh, liquid fraction rumen fluid was squeezed from the rumen contents into a 50-ml conical centrifuge tube (Falcon™, Fisher Scientific, UK). Parasites were removed from the rumen tissue wall into centrifuge tubes using forceps (approximately 40 parasites per tube) and tubes placed into water at 39 °C in an insulated box. Fresh liquid fraction rumen fluid was collected from a freshly opened bovine rumen in the abattoir. Rumen contents were strained through muslin cloth into a pre-warmed 1.5-l thermos flask until almost full. The flask was loosely sealed and both rumen fluid and parasites were transported directly to the laboratory.

In the laboratory, rumen fluid was poured into a large conical flask and placed in a water bath at 39 °C. Fluid was continuously bubbled with nitrogen to maintain anaerobic conditions. Rumen fluid was dispensed in 1 ml of aliquots into individual 7-ml screw top sample tubes (Fisher Scientific, UK) containing the volume of anthelmintic stock solution required to generate the correct experimental concentrations. Sample tubes also contained approx. 0.01 g of finely chopped grass hay as a fermentation substrate for the rumen fluid microbes. A single live rumen fluke was added to each tube and tubes flushed with nitrogen before sealing and placing in an incubator at 39 °C. Rumen fluke motility was scored (Table 1) at 2, 4, 6, 8, 12 and 24 h. At 24 h, the rumen fluid and rumen fluke parasites were removed and separately snap frozen for storage at −80 °C. PCR was used to confirm selected samples as *C. daubneyi* as previously described (Gordon et al. 2013; Huson et al. 2018; Jones et al. 2017).

### Determination of anthelmintic uptake by parasites in vitro

From a representative high, mid and low concentration for each of the 3 anthelmintic compounds tested, 3 adult *C. daubneyi* from the 5 in each group were selected randomly for each treatment group from both DMEM and rumen fluid maintenance conditions. The selected concentrations used for each anthelmintic were as follows: oxyclozanide—25, 15, 5 μM; praziquantel—200, 100, 15 μM; closantel—75, 25, 5 μM. Anthelmintic compounds were subsequently extracted from each parasite as follows: 2 × 0.5-cm stainless steel beads were added to individual rumen fluke in 2-ml microcentrifuge tubes then placed in liquid nitrogen to snap freeze. Samples were bead beaten for 1 min using a Qiagen TissueLyser LT (Qiagen, UK) at full speed. These steps were repeated once more before tubes were centrifuged at 8000×*g* for 2 min. Tubes were then placed in a sonicating water bath for 5 min and vortex mixed for 1 min, before sonication for a further 3 min. A total of 1.55 ml of HPLC-grade acetonitrile was then added to each tube and samples vortex mixed for 1 min and sonicated for 3 min. Samples were placed on ice between sonication and the vortex and sonication steps were repeated a total of 3 times. Samples were then centrifuged at 10,000×*g* for 5 min and the supernatant was removed to a new 2-ml microcentrifuge tube. The supernatant samples were then dried in a vacuum centrifuge. Dried pellets were resuspended in 100-μl mobile phase (70% methanol, HPLC grade) and centrifuged at 10,000×*g* to pellet any small debris before the supernatant was pipetted into 300-μl micro-vials (VWR, UK) for HPLC analysis. This protocol was adapted from those described by Alvarez et al. (2004), Moreno et al. (2014) and Alvarez et al. (2007).

All anthelmintic samples were analysed as previously reported (Davis et al. 2020) by reverse-phase HPLC with online photodiode array detection and electrospray ionisation-ion trap tandem mass spectrometry (HPLC-PDA-ESI/MS^*n*^). Analyses were performed on a Thermo Finnigan LC-MS system (Thermo Electron Corp, Waltham, MA, USA) comprising a Finnigan PDA Plus detector, a Finnigan LTQ linear ion trap with an ESI source and a Waters C_18_ Nova-Pak column (3.9 × 100 mm, particle size 4 μm), with column oven temperature maintained at 30 °C. The PDA scan range was set to 240–400 nm, and injection volume was typically 10 μl. The mobile phase consisted of water/formic acid 100:0.1 (*v*/v; solvent A) and methanol/formic acid 100:0.1 (v/v; solvent B). The column was equilibrated with 95% solvent A at a flow rate of 1 ml min^−1^, with 10% going to the mass spectrometer and the percentage of solvent B increased linearly to 65% over 60 min. MS parameters were as follows: sheath gas 30 and auxiliary gas 15 (both arbitrary units), spray voltage −4.0 kV in negative and 4.8 kV in positive ionisation mode, capillary temperature 320 °C, capillary voltage −1.0 V and 45 V, respectively, and tube lens voltage −68 and 110 V, respectively.

Total peak areas were extracted from the MS data for oxyclozanide and praziquantel and from UV spectra for closantel, owing to this providing the clearest spectral profile for this compound. Data from parasite specimens from negative control treatments and rumen fluke which had been spiked with a known amount of each anthelmintic during the extraction process were used to generate standard curves, and detectable anthelmintic concentrations within each exposed experimental parasite sample were calculated. Differences in detected anthelmintic mass between the parasite maintained in either DMEM or rumen fluid were analysed for significance in PAST (Hammer et al. 2001) using a Students *T* test at each different anthelmintic concentration analysed by HPLC.

### Determination of visible damage to parasite tegument or morphology after anthelmintic exposure

The highest anthelmintic concentration treatments of oxyclozanide and praziquantel observed to have a negative effect on parasite motility in both the DMEM and rumen fluid experiments were selected for scanning electron microscopy (SEM) analysis. Along with anthelmintic-negative solvent controls and controls preserved immediately following collection from the host, individual rumen flukes were selected at random for examination under SEM to observe if any visible changes in morphology had occurred in relation to each treatment. In brief, following parasite collection (control) or in vitro maintenance in DMEM or rumen fluid (oxyclozanide, praziquantel and solvent control specimens), three individual parasites from each selected treatment group were transferred to 1.5 ml of fixing solution; 4 °C 2.5% v/v glutaraldehyde in 0.1 M sodium cacodylate at pH 7.2, in a 2-ml microcentrifuge tube, and stored at 4 °C until required. Rumen fluke specimens were then prepared for SEM as previously described (Crusco et al. 2018) before being attached to self-adhesive conductive carbon tabs on 0.5″ aluminium specimen stubs (both Agar Scientific, Stanstead, UK) and gold coated for 5 min in a PolaronE5000 SEM coating unit. Flukes were then imaged using a Hitachi S-4700 FESEM microscope using the Ultra High-Resolution mode and an accelerating voltage of 3.0 kV, working distance 5.0 mm and images captured at 2560 × 1920 resolution.

## Results

### Anthelmintic screens

**Fig. 1:**
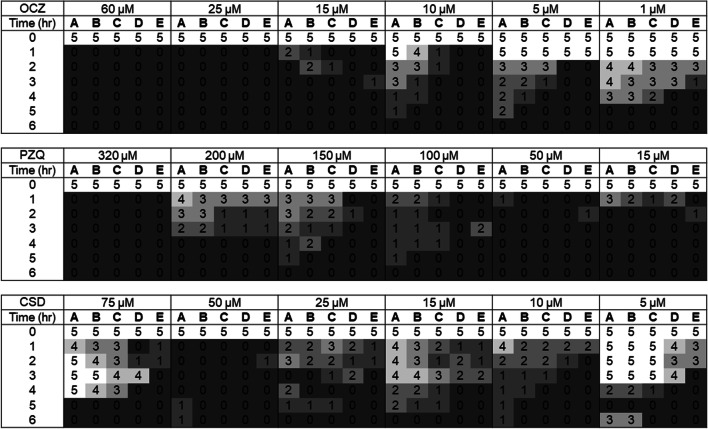
Motility scores for rumen fluke maintained over a 6-h period in DMEM in oxyclozanide (OCZ), praziquantel (PZQ) and closantel (CSD). Five replicate rumen fluke were included per anthelmintic concentration, at 6 different concentrations. Control parasites were maintained in the same conditions in the presence of the highest DMSO solvent concentration needed to add each anthelmintic to the DMEM. Control parasites maintained a motility score of 5 throughout (data not shown)

The three anthelmintic compounds selected to test in the DMEM and rumen fluid screening methods all showed activity against parasite motility scores when maintained in DMEM media over a 6-h period compared to the control group maintained in the same volume of DMSO as would be present in the highest concentration anthelmintic treatment, but not exposed to any drugs. In brief, after a 6-h period the rumen fluke exposed to closantel, oxyclozanide and praziquantel all had motility reduced to 0 in all 5 replicates per concentration at concentrations of 15 μM and above (Fig. 1). At 15 μM praziquantel specimens were reduced to a zero score consistently after 3-h exposure in DMEM. The most efficacious anthelmintic in the screen was oxyclozanide, reducing motility scores to 0 at 1-μM concentration after 5 h. All solvent control and negative control motility scores remained at 5 throughout the experiment (data not shown).

**Fig. 2:**
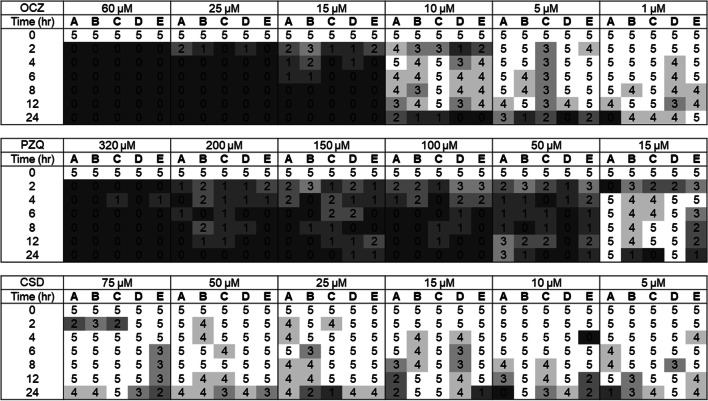
Motility scores for adult rumen fluke maintained over a 24-h period in rumen fluid in oxyclozanide (OCZ), praziquantel (PZQ) and closantel (CSD). Five replicate rumen fluke were included per anthelmintic concentration, at 6 concentrations. Control parasites were maintained in the same conditions in the presence of the highest DMSO solvent concentration needed to add each anthelmintic to each rumen fluid replicate. The control parasites maintained a motility score of 5 throughout (data not shown)

In contrast, in the rumen fluid in vitro environment, closantel failed to replicate the reduction in motility to a score of 0, as was seen in the DMEM media after 6 h, even after the extended 24-h maintenance period (Fig. 2). Although some reduction in parasite motility was observed for all closantel treated groups in the rumen fluid screen, regardless of concentration, this reduction in motility was only observed to average scores of 2.8 on the 5–0 scale. However, oxyclozanide and praziquantel were observed to stop all motility of the rumen fluke during the course of the 24-h period in rumen fluid. For praziquantel, an estimated concentration of 75 μM or above was required to largely reduce motility scores to 0, although not all individuals were immotile at this point. A concentration of 320 μM praziquantel appeared effective, with all individuals consistently scoring 0 for motility from 6 h of in vitro exposure onwards. Oxyclozanide was also effective, with a 25-μM concentration required to reduce motility to 0 in all 5 individual rumen fluke replicates after just 4 h of in vitro exposure. As with DMEM, all solvent and negative control parasite motility scores remained at 5 throughout the experiment (data not shown).

### Analysis of rumen fluke anthelmintic uptake during in vitro screening via HPLC-MS

**Fig. 3:**
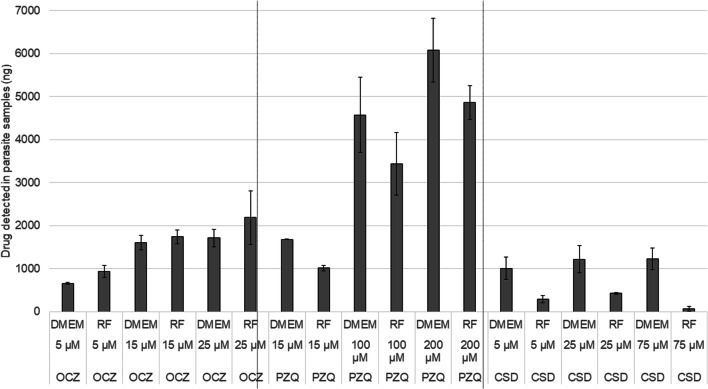
Anthelmintic levels detected in parasite somatic tissues using HPLC. RF or DMEM denotes rumen fluid or DMEM screening protocol; anthelmintic concentrations are shown in micromolar, and the extracted detected anthelmintic mass shown as an average for the 3 individual parasites within each treatment group in ng. No compounds of the same mass as each tested anthelmintic could be detected in anthelmintic-negative parasite samples subjected to the same processing protocol on the HPLC platform. Error bars show the standard error of the mean for each group of replicates

A high-pressure liquid chromatography (HLPC) assay was used to determine if the rumen fluke parasites actually internalised the anthelmintic compounds they were exposed to in vitro. To this end, three individual parasites from a low, mid and high tested concentration of each anthelmintic in both DMEM and rumen fluid screening conditions were randomly selected for extraction from the parasite somatic tissues followed by analysis with HPLC. Notably, for both closantel and praziquantel, higher anthelmintic levels were detected within the DMEM-maintained parasites than in the rumen fluid-maintained parasites, despite the 18-h difference in the in vitro maintenance time endpoints, and therefore longer period of exposure (Fig. 3). In the case of oxyclozanide, higher levels were detected in the parasites screened in rumen fluid compared to their counterparts in the same concentrations in DMEM. Anthelmintic levels extracted from the parasites generally increased with the in vitro anthelmintic concentration, although in the 75 μM closantel samples from the rumen fluid screen only an average of 67.8 ng of anthelmintic was detected, lower than the 5 μM and 25 μM conditions within this treatment. It is interesting to note that despite considerably longer maintenance and exposure time in rumen fluid than DMEM (24 h instead of 6 h), only the oxyclozanide treated specimens had a higher level detectable in the rumen fluid screened parasites compared to the corresponding DMEM treatment. For the parasites exposed to closantel which were motile at the end of the 24-h rumen fluid screen, minimal levels of anthelmintic were detected in the somatic preparations by HPLC. Additionally, a higher concentration of praziquantel was required to see similar activity against rumen fluke in the rumen fluid in vitro conditions as opposed to in DMEM. Closantel treatments were ineffective at reducing fluke motility to 0 in rumen fluid, despite their apparent success in the DMEM screening.

Statistical analysis of the level of detectable anthelminticobserved in the DMEM and rumen fluid-maintained parasite samples did not identify significant differences between the two screening approaches, except for the comparison between the cultures containing 75 μM closantel, showing a significantly greater apparent anthelmintic uptake in the DMEM maintenance conditions compared to maintenance in rumen fluid (*p* = 0.009). However, it was not possible to account for the different anthelmintic exposure times in this instance. Despite a lack of statistical significance, there was a clear trend towards greater resilience of parasites exposed to praziquantel and closantel in the rumen fluid screening approach.

### SEM imaging of parasites following in vitro anthelmintic exposure

**Fig. 4:**
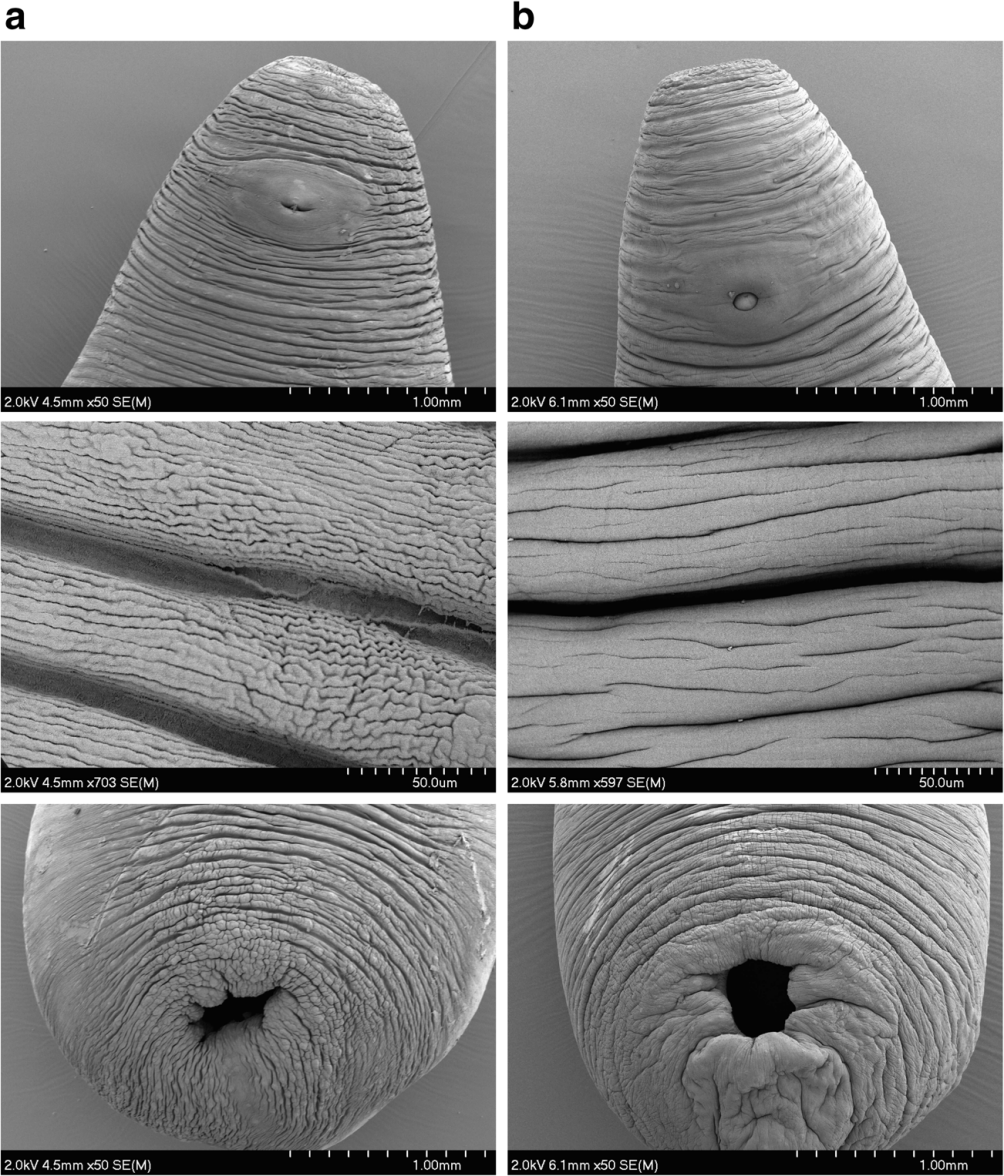
SEM comparison of adult *C. daubneyi* maintained in DMSO/DMEM and rumen fluid. **a** DMSO/DMEM 6-h DMEM-maintained rumen fluke. Top: anterior end showing oral sucker and genital pore. Mid: Grooves and folds of the mid-body tegument. Bottom: Posterior sucker used for attachment to rumen wall/papillae. **b** Rumen fluid 24-h maintained rumen fluke. Top: anterior end showing oral sucker and genital pore. Mid: Grooves and folds of the mid-body tegument. Bottom: Posterior sucker used for attachment to rumen wall/papillae

To examine any morphological changes in response to anthelmintic exposure, and any differences in such effects between the DMEM and rumen fluid maintenance approaches, rumen fluke specimens were examined under SEM. Individual fluke (motility ‘0’) from 25-μM oxyclozanide and 320-μM praziquantel treatments were fixed for SEM imaging. Flukes not exposed to the DMSO solvent during in vitro maintenance and specimens preserved immediately following collection from the host were also prepared for SEM as untreated controls. Images of control fluke from both DMEM and rumen fluid screening conditions showed no apparent damage or disturbance to the parasite tegument (Fig. 4a, b).

**Fig. 5:**
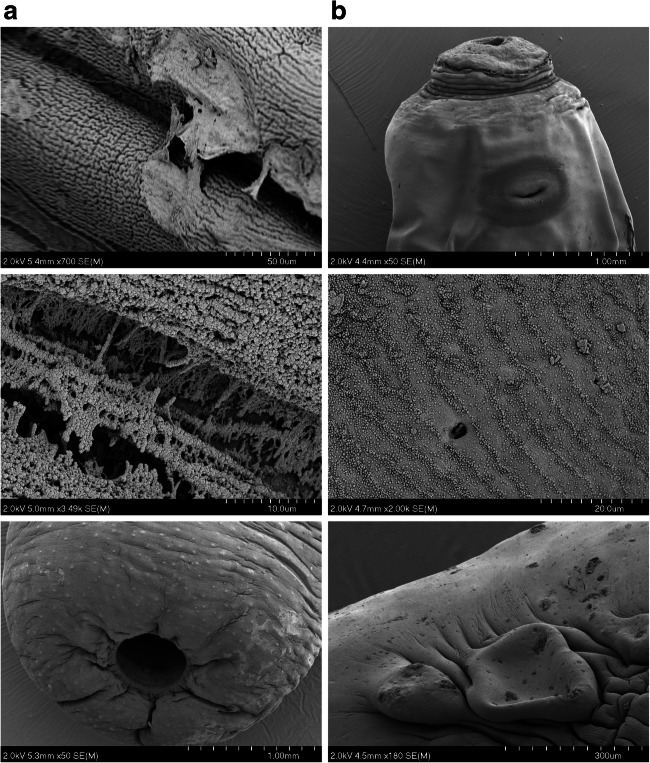
SEM comparison of adult *C. daubneyi* maintained in DMSO/DMEM and rumen fluid in the presence of 25uM oxyclozanide. **a** Oxyclozanide, 25 μM, 6-h DMEM-maintained rumen fluke. Top: blebbing and shedding of the outer tegumental layers seen on the mid-body. Mid: tearing between the tegument grooves. Bottom: Swelling of the tissue of the posterior sucker. **b** Oxyclozanide, 25 μM, 24-h rumen fluid-maintained rumen fluke. Top: anterior end showing oral sucker and genital pore. Mid: holes visible in the tegument around the genital pore. Bottom: swelling and blebbing of the tissue at the posterior sucker

Images of fluke maintained for 6 h in DMEM containing 25 μM oxyclozanide revealed evidence of blebbing, shedding and tearing of the upper layers of the tegument (Fig. 5a, b) along with swelling and loss of definition to the tegument grooves around the posterior and oral suckers. Specimens maintained in 25 μM oxyclozanide over 24 h in rumen fluid were visibly in poor condition prior to SEM sample preparation. When imaging these samples on the SEM significant morphological changes could be observed with a loss of the definition of the tegumental grooves and folds compared to control samples, with also ‘wrinkling’ and collapse of the body structure. Moreover, apparent holes were observed in genital pore tissue, with blebbing and peeling on the tegument in the mid-region of the parasite body and swelling, loss of definition and blebbing also noted on the tegument around the posterior sucker.

**Fig. 6:**
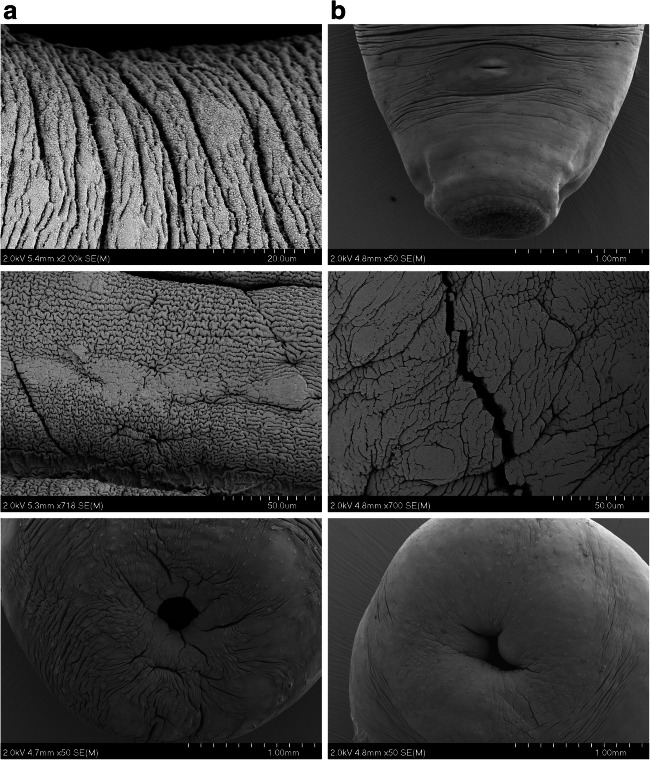
SEM comparison of adult *C. daubneyi* maintained in DMSO/DMEM and rumen fluid in the presence of 320uM praziquantel. **a** Praziquantel, 320 μM, 6-h DMEM-maintained rumen fluke. Top: some tearing of the tegument around the genital pore. Mid: swelling and loss of definition in the tegument folds. Bottom: Swelling and loss of definition to the tissue around the posterior sucker. **b** Praziquantel, 320 μM, 24-h rumen fluid-maintained rumen fluke. Top and bottom: significant swelling of the parasite body at the oral and posterior suckers. Mid: cracking observed in the outer tegument near to the posterior sucker

The fluke maintained in 320 μM praziquantel for 6 h in DMEM showed swelling to the tissue around the posterior sucker (Fig. 6a), as well as swelling and loss of definition in the tegument folds around the oral sucker, swelling of the tegument in the mid-section and some tearing of the tegument starting to occur around the genital pore. In the fluke maintained for 24 h in rumen fluid and praziquantel at 320 μM swelling of the parasite body was obvious, particularly around the posterior sucker (Fig. 6b). There was a loss of definition to the tegumental grooves and folds which can be seen on the control specimens. Several areas of tegument blebbing were seen close to the oral sucker, and tegumental tearing and cracking were observed near to the caudal sucker, along with pronounced swelling of the tissue here.

## Discussion

Relevant in vitro compound screening tools are required to support control of existing and emerging pathogens. This study has identified a rumen fluid based in vitro screening tool for screening new candidates or repurposing existing compounds for adult rumen paramphistome parasites. The standard DMEM in vitro screen, an optimised system for related trematode species (Morphew et al. 2014), suggested that all three test anthelmintic compounds have potential activity against adult rumen fluke, as all were associated with a notable reduction in motility in comparison to the anthelmintic-free controls after 6 h. However, a rumen fluid screen that better mimics the host rumen environment demonstrated that only 2 compounds, oxyclozanide and praziquantel, were active against adult rumen fluke. Indications from motility scoring at the higher tested concentrations (above 15 μM for oxyclozanide and at 320 μM for praziquantel) were that both these anthelmintics caused the death of these parasites in both the DMEM and rumen fluid conditions, as supported by the visible damage to the parasites, and morphological changes observed under SEM in comparison to anthelmintic-free control specimens and those preserved directly after collection from the host. The higher levels of oxyclozanide within rumen fluid-maintained fluke bodies vs their DMEM-maintained counterparts may be explained as oxyclozanide caused rapid death of the fluke in DMEM (within 1 h for the 25-μM treatment), allowing comparatively little time for them to take up the drug, whereas in rumen fluid, it was observed that a higher concentration of oxyclozanide was required to produce the comparable effects and only parasites exposed to >15 μM drug concentrations consistently appeared dead after the 24 h in vitro maintenance period. Alternatively, the longer exposure period in the rumen fluid treatment may have allowed greater levels of anthelmintic to passively accumulate in the parasite tissue following death, when fluke would be unable to excrete these compounds. It was not possible to determine the exact mechanism of anthelmintic accumulation in parasite tissues during this study. Higher levels of internalised anthelmintic were observed in both closantel- and praziquantel-treated parasites maintained in DMEM, where it is considered that the parasites would have been under greater environmental stress, whereas the lower levels detected in the specimens maintained in rumen fluid suggests that parasites are more resilient against anthelmintic toxic effects in a more favourable environment.

Similar effects of anthelmintic compounds on rumen parasites have previously been observed using SEM. Examination of gastrointestinal parasites including rumen flukes after plant compound or anthelmintic exposure using SEM imaging has been widely used to assess signs of physical damage to the parasites caused by the compounds in question (Martínez-Ortíz-de-Montellano et al. 2013; Saowakon et al. 2013). However, these previous studies have been completed using less environmentally relevant in vitro screens.

The activity of praziquantel reported here against adult *C. daubneyi* must currently be considered with caution. Previous in vivo studies have found no significant success of praziquantel in treating infections with other species of rumen fluke (Pakharukova et al. 2015).

Predicting in vivo activity from in vitro anthelmintic trials based on motility scoring has been deemed problematic (Behnke et al. 2008; Pakharukova et al. 2015; Saowakon et al. 2013) as motility scoring is a relatively subjective measure of death. Inactivity is often presumed to indicate parasite death although this may not necessarily be the case, particularly with praziquantel which is known to cause paralysis in other trematode and cestode parasites (Pakharukova et al. 2015). Therefore, steps should be taken to support any such assumption with other data where possible. For large parasites such as rumen fluke, staining for cell death and microscopic examination is not suitable (Pakharukova et al. 2015), and high throughput screening methods looking at phenotypic indicatiors of parasites succumbing to treatment that have been developed and applied to other helminths (Buckingham et al. 2014) are not currently available for paramphistomes. Hence, the decision to incorporate SEM and HPLC steps into our anthelmintic discovery pipeline.

This report highlights the importance of using relevant in vitro screens. Although the predicted calculations of ‘in vivo’ dosage concentrations used at this stage of discovery cannot predict factors such as the balance of solid and liquid fraction rumen contents, drug dilution due to uptake by host tissues, and any possible dilution due to uptake by rumen microbes, methods which can minimise potential ‘false-positive’ results (as could be indicated if the results of the DMEM screen with closantel were considered in isolation) are valuable to help direct more in-depth future studies, including in vivo trials. It was also not possible to account for the effect of digesta flow and dilution rate on compounds moving out of the rumen or the effect of individual animal variation due to age, breed, etc. on the composition of rumen fluid during this in vitro screen, although it was ensured that all treatments used the same pooled rumen fluid source to remove such variability within this experiment. As with all in vitro work, further validation of any conclusions should be conducted during in vivo trials where possible and appropriate. Effective concentrations observed in vitro are frequently not comparable to effective doses identified in vitro (Githiori et al. 2006), highlighting the need to extend results from in vitro work with confirmation of efficacy and required dose through follow-up in vivo experiments.

There are previous positive reports of closantel efficacy against rumen fluke, with a reduction in FEC scores reported in cattle (Arias et al. 2013), although a separate study in sheep did not identify any efficacy of closantel for treating paramphistome infections (García-Dios et al. 2020). This is supported by our in vitro findings, wherein a more favourable rumen fluid environment closantel was not found to show significant activity against adult *C. daubneyi* and was not associated with apparent parasite death over a 24-h period in concentrations up to 75 μM.

The work described here clearly highlights the relevance of the rumen fluid as a medium for performing in vitro screens with adult *C. daubneyi* in addition to alternative species of rumen fluke. From the current work, DMEM, or likely equivalents, are not appropriate and have the potential to indicate false-positive compound efficacy. This is likely to apply to other simple media which are not optimised for supporting rumen fluke in vitro. The approach described using liquid fraction rumen fluid could easily be adapted to the screening of other paramphistome parasites in tropical areas where there is an urgent need for the identification of suitable anthelmintic products due to the significant morbidity and mortality associated with paramphistomosis locally (Shaheen et al. 2013).

## Data Availability

Available.

## Materials availability

Available.
